# Author Correction: A mechanism for CO regulation of ion channels

**DOI:** 10.1038/s41467-018-05622-6

**Published:** 2018-08-17

**Authors:** Sofia M. Kapetanaki, Mark J. Burton, Jaswir Basran, Chiasa Uragami, Peter C. E. Moody, John S. Mitcheson, Ralf Schmid, Noel W. Davies, Pierre Dorlet, Marten H. Vos, Nina M. Storey, Emma Raven

**Affiliations:** 1 Department of Chemistry and Leicester Institute of Structural and Chemical Biology, University of Leicester, Leicester, LE1 7RH England; 2 Department of Molecular and Cell Biology and Leicester Institute of Structural and Chemical Biology, University of Leicester, Leicester, LE1 9HN England; 3 Institute for Integrative Biology of the Cell (I2BC), CEA, CNRS, Univ. Paris-Sud, Université Paris-Saclay, 91198 Gif-sur-Yvette cedex, France; 4 LOB, Ecole Polytechnique, CNRS, INSERM, Université Paris-Saclay, 91128 Palaiseau Cedex, France

Correction to: *Nature Communications* 10.1038/s41467-018-03291-z; published online 02 March 2018

The originally published version of this article contained an error in the subheading ‘Heme is required for CO-dependent channel activation’, which was incorrectly given as ‘Hame is required for CO-dependent channel activation’. This has now been corrected in both the PDF and HTML versions of the Article.

