# Supplementary material for: A mechanism for CO regulation of ion channels
Source: Nat Commun. 2018 Mar 2;9:907. doi: 10.1038/s41467-018-03291-z (PMC5834611; doi:10.1038/s41467-018-03291-z)
Supplement: Supplementary file 1 — Supplementary material [file 41467_2018_3291_MOESM1_ESM.pdf]

a

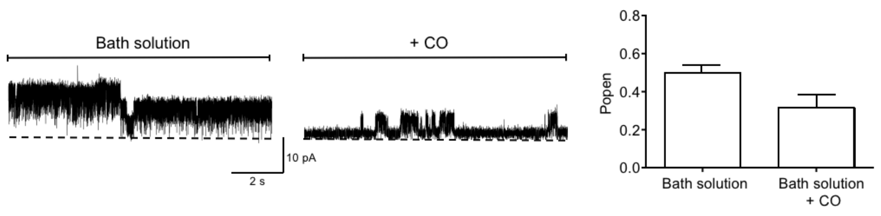

b

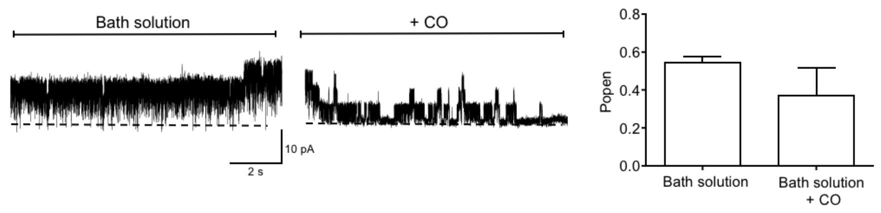

Supplementary Figure 1. Application of CO without the prior application of heme fails to increase K_{ATP} channel activity.

(a) Wild type channels were perfused with a control physiological buffer solution and under conditions where only CO was added without prior application of heme (or ATP), there was no increase in average Popen observed. This is in contrast to the increase in channel activity seen when CO was added in the presence of heme (compare **Fig. 1b (top)).** It is worth noting that a proportion of the cells (5/8) showed a decrease in Popen in response to application of CO alone while the other 3/8 cells showed no change and on average there was no significant difference (P>0.05, paired T Test).

(b) K_{ATP} channels expressing the triple C628S/H631A/H648A mutant (in which the heme binding site is removed) showed no significant change in average K_{ATP} channel activity (n=4, P>0.05, paired T Test). Without prior addition of heme, an increase in Popen was not observed upon addition of CO (compare **Fig. 1b (bottom))**. It is worth noting that there was no significant change in the average Popen, however half cells tested showed a large decrease in response to CO application whilst the other half of the cells tested showed no change in activity. In the absence of prior application of heme, any decrease in K_{ATP} activity observed with CO alone indicates that the effects of CO must be due to interaction with the channel at a site that is independent of the SUR2A heme-binding binding motif, which is consistent with the idea that there is more than one mechanism for CO regulation in cells.

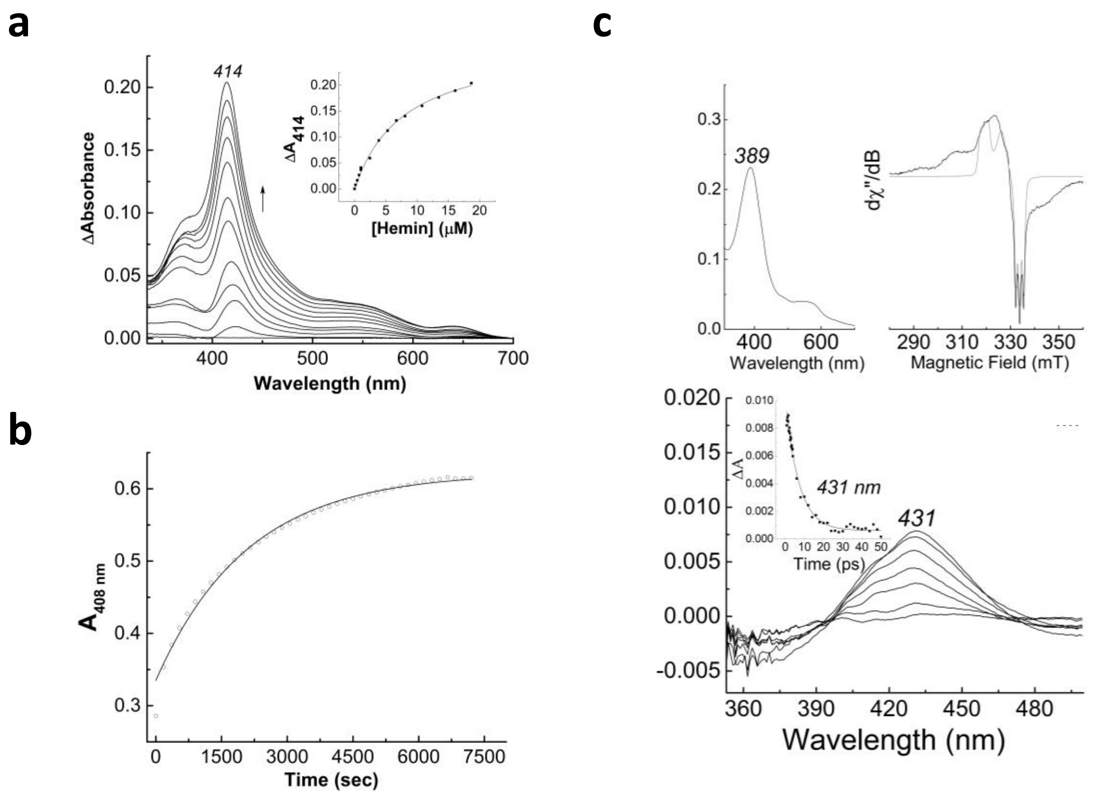

Supplementary Figure 2. Quantification of SUR2A interaction with heme and ligands.

(a) Difference spectra observed during titration of SUR2A$^{615-933}$ with ferric heme. Inset shows hyperbolic fitting to a 1:1 binding equation (K_d = 8.0 ± 0.6 μM).

(b) Plot of absorbance at 408 nm (reporting on formation of heme-bound myoglobin) on reaction of apo-myoglobin (36 μM) with heme-SUR2A$^{615-933}$ complex (6.4 μM). The observed first-order rate constant for heme dissociation, $k_{off\ (heme)}$, is 4.8 ± 0.1 x 10^{-4} s^{-1}.

(c) (Top) Left: Absorption spectrum of ferrous heme-SUR2A$^{615-933}$ in complex with NO; the spectrum is consistent with a 5-coordinate NO-bound species, as observed in other heme systems[21,107-109]. Right: X-band EPR spectrum of ferrous heme-SUR2A$^{615-933}$ in complex with NO and the best-fit simulation (g values 2.100 ± 0.010, 2.033 ± 0.010 and 2.008 ± 0.005; ^{14}NO nitrogen hyperfine coupling 50 ± 5, 67 ± 10 and 45 ± 5 MHz). These g-values and hyperfine coupling constants are consistent with other five-coordinate ligand-bound complexes in other heme proteins[106,110]. **(Bottom)** Transient absorption spectra observed on exposure of the heme-NO-SUR2A$^{615-933}$ to a femtosecond laser pulse, at various delay times (1.8, 3, 4, 6, 8, 18 and 50 ps). The ligand dissociates and the figure shows transients at 431 nm for recombination of the ligand. The spectra are dominated by an increased absorption at 431 nm and bleaching of the ground state 380 nm transition. Global analysis reveals a very fast component assigned to excited state photophysics[111,112] and a 7-ps component assigned to geminate ligand recombination. Fitting of the kinetic trace at 431 nm reveals that only a very small fraction (~6%) of the NO does not rebind within 50 ps. The remainder of the NO (94%) rebinds in a single exponential phase. These features are similar to other 5-coordinate heme-NO complexes (**Supplementary Table S6**).

Supplementary Table 1: Association (k_{on}) and dissociation (k_{off}) rate constants, and equilibrium constants (K_d), for heme binding to SUR2A and other heme proteins

Protein	k_{on} ($M^{-1}s^{-1}$)	k_{off} (s^{-1})	K_d (M)	References
SUR2A$^{615-933}$	0.6×10^{2} *	4.8×10^{-4}	8×10^{-6}	This work
SOUL	2.5×10^{5}	1.2×10^{-3}	4.8×10^{-9}	[1]
p22HBP	2.1×10^{8}	4.4×10^{-3}	2.1×10^{-11}	[1]
Sperm whale Mb	7.0×10^{7}	8.4×10^{-7}	1.2×10^{-14}	[2,3]
Sperm whale Mb H93G	7.0×10^{7}	1.2×10^{-2}	1.6×10^{-10}	[2,3]
Hb	2.9×10^{7}	7.1×10^{-6} (α)	2.5×10^{-13}	[2-4]
		9.4×10^{-4} (β)	3.2×10^{-11}	[2,3]
BSA	5.7×10^{7}	1.1×10^{-2}	2.2×10^{-10}	[2]
mPer2-PAS-A	3.5×10^{7}	6.3×10^{-4}	1.8×10^{-11}	[5]
NPAS2-bHLH-PAS-A	3.3×10^{7}	5.3×10^{-3}	1.6×10^{-10}	[6]
HRI	1.1×10^{7}	1.5×10^{-3}	1.4×10^{-10}	[7]

* Calculated from $K_d = k_{off}/k_{on}$.

IV. Supplementary Table 2. Comparison of absorption maxima for various heme proteins[a]

Protein	Fe(II)	Fe(II)-CO	References
SUR2A$^{615-933}$	385, 425, 531, 559, 577	420, 540, 569	This work
heme	383, 550, 574	407, 537, 567	This work
BK channel, HBD	426, 530, 560	419, 535, 568	[8]
O_2 binding proteins			
Sperm whale myoglobin	434, 556	423, 542, 579	[9]
E. coli DOS	427, 532, 563	423, 540, 570	[10,11]
FixL*	433.5, 565	425, 545, 576	[12,13]
SOUL	422, 527, 558	418, 536, 563	[1]
P22HBP	422, 530, 560	416, 537, 567	[1]
Catalytic enzymes			
Nitric oxide synthase	412, 560	444	[14]
$P450_{cam}$	409, 542	448, 550	[15]
Chloroperoxidase	408, 552	445, 550	[15]
Regulatory proteins			
E75	425, 530, 558	421, 540, 569	[16]
Rev-erb β	428,533,558	421, 540, 568	[16]
RcoM-2	425, 532, 562	423, 540, 570	[17]
CooA	425, 530, 559	422, 540, 569	[18,19]
Cystathione β synthase	450	422	[20]
Heme-regulated eIF2α	428, 530, 560	423, 540, 570	[21]
PAS domain proteins			
Clock PAS-A	423, 527, 559	420, 540, 570	[22]
NPAS2-PAS-A	423, 530, 558	420, 530, 568	[6]
NPAS2-PAS-B	424, 529, 557	420, 536, 571	[23]
		426, 530, 561	[24]
mPer2-PAS-A	425, 529, 558	420, 539, 565	[5]

[a] Reviewed also in reference [25].

V. Supplementary Table 3: Association (k_{CO}) and dissociation (k_{-2}) rate constants, and equilibrium constants (K_d), for CO binding to SUR2A and other heme proteins

Protein	k_{CO} ($M^{-1}s^{-1}$)[a]	k_{-2} (s^{-1})[a]	K_d (μM)	k_1 (s^{-1})[a]	References
SUR2A[615-933]	0.17-0.37 x 10^6	0.05	0.6	20-30	This work
A. thaliana Hbs[b]	0.55-50 x 10^6	0.0012-0.0013		34/43	[26-28]
A.thaliana GLB3	0.014 x 10^6	0.001			[27]
Tomato SOLly GLB1	1 x 10^6	0.02	0.02	200	[29]
Oryza sativa Hb1	7.2/6.8 x 10^6	0.001	0.00014	40	[30-32]
Oryza sativa Hb2	1.8 x 10^6			15	[31]
Synechocystis Hb	90 x 10^6			14	[31,33]
Human Ngb	50/65 x 10^6	0.014	0.00021	0.6/4.5	[28,34]
Murine Ngb	55/72 x 10^6	0.013	0.00018	0.5-0.6/1.2	[28,34-36]
Human Cgb	5.6 x 10^6	0.003	0.0217	~0.5/0.09	[31,37]
Barley Hb	0.57 x 10^6	0.0011	0.00193		[38]
Drosophila Hb	13 x 10^6			30/40	[28,39]
Hb α–chain R/β-chain R	2.9/7.1 x 10^6	0.0046/0.0072			[40]
Sperm whale Mb	0.51 x 10^6	0.019	0.037		[40] [40]
A.xylosoxidans cytc'	101 x 10^6	0.028	280		[41]
cytc mutants				70	[42]
E. coli DOS, full-length	0.00081/4 x 10^6	0.0025/0.007	3.1	2/9	[43,44]
E. coli DOS, PAS domain	0.0078/5/0.0011 x 10^6	0.0045/0.011	0.58/10	400	[44-46]
B. japonicum FixLH	0.005 x 10^6	0.045	9		[47]
R.meliloti FixLH	1.7x10^{-4}	0.083			[47]
sGC	3.6 x 10^4	3.5	98		[48]
AxPDEA1	0.21 x 10^6	0.058	0.28		[49]
Fe(PPIX)(2-MeIm)(CO)	1.6 x 10^6				[45]

[a] k_{CO}, k_1 and k_{-2} are defined or derived from Scheme 1 in the main text. k_1 is the first-order rate constant for dissociation of a distal protein ligand in a 6-coordinate heme species (also defined Eq 1), which can be extracted from kinetic data for CO binding.
[b] There are two types of *A. thaliana* Hb (*A. thaliana* Hb1 and *A. thaliana* Hb2), so we report a range of reported values in the table.

VI. Supplementary Table 4. Comparison of high frequency vibrational modes of CO-bound complexes of heme proteins.

Protein	ν_4	ν_3	ν_2	References
SUR2A[615-933]	1370	1498	1584	This work
Heme	1372	1501	1589	This work
horseMb	1373	1501	1585	[50]
E. coli DOS	1370	1496	1581	[51]
SOUL	1371	1499	1582	[1]
p22HBP	1372	1499	1583	[1]
E75(LBD)	1367	1493	1579	[16]
Reverbβ(LBD)	1368	1493	1581	[16]
CooA	1371	1493	1579	[52,53]
HemAT-Bs	1368	1495	1578	[54]
sGC	1370	1499	1584	[55]
NPAS PASA	1372	1497/1468	1583/1556	[56]
NPAS2-PASB	1372	1498/1467	1583/1558	[23]
mPer2 PASA	1370	1465/1497	1554/1586	[5]

VII. Supplementary Table 5. Comparison of ν(Fe-CO) and ν(C-O) stretching frequencies of various heme proteins.

Protein	ν(Fe-C)	ν(C-O)	References
SUR2A$^{615-933}$	494	1965	This work
Sperm whale Mb	512	1944	[57]
Human HbA	507	1951	[57]
HRP form II	537	1904	[58,59]
form III	516	1934	[60]
CCP form II	530	1922	[61]
form II’	503	1948	[61]
CCPMI (alk)	503	1922	[62]
Mtb. KatG-WT form I	522	1926	[63]
Mtb. KatG-WT pH=7.2	525	1923	[64]
pH=10	520	1928	[64]
HemAT	494	1964	[54]
E. coli DOS	486	1973	[51,65]
FixL	498	1962	[66]
DosS	490	1971	[67]
NPAS2-PAS-A	491	1962	[56]
$GAF_{All4978}$	514	1957	[68]
CBS	497	1961	[20]
CooA	487	1982	[69]
	487	1969	[70,71]
sGC_1	497	1959	[72,73]
sGC_2	473	1987	[74,75]
HRI	492	1967	[21]
HRI-NTD	494	1963	[21,76]
HRI-NTD	493	1960	[77]
NP4	499	1962	[78]
	513	1950	
Rev-erbβ(LBD)	500	1948	[79]
	515	1948	[79]
E75(LBD)	494	1958	[79]
cytochrome c’	491	1966	[80]
PPDMe(ImH)	495	1960	[58]
nNOS, substrate-free	487	1949	[81]
nNOS, substrate-free	501	1930	[81]
nNOS + Arg	503	1929	[81]
$iNOS_{FL}$, substrate-free	487	1945	[82]
$iNOS_{FL}$ + Arg	512	1906	[82]
$iNOS_{oxy}$, substrate-free	491	1946	[83]
$iNOS_{oxy}$ +Arg	512	1907	[83]
$iNOS_{oxy}$ +H_4B	490	1944	[83]
$iNOS_{oxy}$ +Arg/H_4B	512	1905	[83]

CYP101, substrate-free	464	1963	[84-87]
CYP101 + norcamphor	473	1947	[87]
CYP101 + adamantanone	474	1942	[87,88]
CYP101 + camphoroquinone	476	1941	[87]
CYP101 + fenchone	480	1945	[87]
CYP101 + camphor	481	1940	[84-87,89,90]
CYP101 + tetramethylcyclohexanone	485	1934	[87]
CYP17, substrate-free	472	1957	[91]
CYP17, substrate-free	485	1946	[91]
CYP17 + 17-OH pregnenolone	491	1938	[91]
CYP17 + 17-OH pregnenolone	505	1928	[91]
CYP17 + 17-OH progesterone	491	1938	[91]
CYP17 + progesterone	498	1932	[91]
CYP17 + pregnenolone	498	1940	[91]
TPP(OH)DMF	527	1947	[92]
TPP(NH_2)/DMF	527	1948	[92]
TPP(OCH_3)/DMF	526	1949	[92]
TPP/DMF	525	1951	[92]
TSMP/H_2O	527	1957	[93]
TPivP/THF	526	1957	[94]
TPP(CN)/DMF	521	1957	[92]
TPP(2,6-difluoro)/DMF	521	1963	[92]
TPP(2,6-dichloro)/DMF	516	1966	[92]
TPP/Bz	524	1973	[95]
BSA	526	1962	[1]
HSA	524	1960	[96]
heme in PBS, pH 6.9	525	1962	[96]
Heme (80% glycerol)	530	1955	[97]
heme in Hepes, pH 7.5	525	1948	This work

VIII. Supplementary Table 6: Parameters of ligand recombination in heme proteins.

Protein	Recombination time constants / ps (rebinding fraction)	Non-decaying phase	References
SUR2A$^{615-933}$	22 (14%), 150 (26%), 2500 (50%)	10%	This work
DNR	100 (30%), 900(35%)	35%	[98]
CooA	78(60%), 386(30%)	10%	[99,100]
E. coli DOS	1500(60%)	40%	[101]
R220H FixL	280(25%), 2400(35%)	40%	[102]
RcoM-2	170(65%), 500(35%)	0%	[103]
NO Binding			
SUR2A$^{615-933}$	7(94%)	6%	This work
sGC	7.5 (97%)	3%	[104]
A.xylosoxidans cyt *c*'	7.0 (100%)	0%	[105]
cytc/CL	7.0 (89%)	11%	[106]

SUPPLEMENTARY REFERENCES

1 Sato, E., Sagami, I., Uchida, T., Sato, A., Kitagawa, T., Igarashi, J., Shimizu, T. SOUL in Mouse Eyes Is a New Hexameric Heme-Binding Protein with Characteristic Optical Absorption, Resonance Raman Spectral, and Heme-Binding Properties. *Biochemistry* **43**, 14189-14198 (2004).

2 Hargrove, M. S., Barrick, D., Olson, J.S. The association rate constant for heme binding to globin is independent of protein structure. *Biochemistry* **35**, 11293-11299 (1996).

3 Hargrove, M. S., Singleton, E.W., Quillin, M.L., Ortiz, L.A., Jr., Olson, J.S. Mathews, A.J. His64(E7)-Tyr apomyoglobin as a reagent for measuring rates of hemin dissociation. *J. Biol. Chem.* **269**, 4207-4214 (1982).

4 Rose, M. Y. & Olson, J. S. The kinetic mechanism of heme binding to human apohemoglobin. *J Biol Chem* **258**, 4298-4303 (1983).

5 Kitanishi, K., Igarashi, J., Hayasaka, K., Hikage, N., Saiful, I., Yamauchi, S., Uchida, T., Ishimori, K., Shimizu, T. Heme-Binding Characteristics of the Isolated PAS-A Domain of Mouse Per2, a Transcriptional Regulatory Factor Associated with Circadian Rythms. *Biochemistry* **47**, 6157-6168 (2008).

6 Mukaiyama, Y. *et al.* Spectroscopic and DNA-binding characterization of the isolated heme-bound basic helix-loop-helix-PAS-A domain of neuronal PAS protein 2 (NPAS2), a transcription activator protein associated with circadian rhythms. *FEBS J* **273**, 2528-2539 (2006).

7 Miksanova, M., Igarashi, J., Minami, M., Sagami, I., Yamauchi, S., Kurokawa, H., Shimizu, T. Characterization of heme-regulated eIF2a kinase:Roles of the N-terminal domain in the oligomeric state, heme binding, catalysis, and inhibition. *Biochemistry* **45**, 9894-9905 (2006).

8 Yi, L., Morgan, J. T. & Ragsdale, S. W. Identification of a thiol/disulfide redox switch in the human BK channel that controls its affinity for heme and CO. *J Biol Chem* **285**, 20117-20127 (2010).

9 Antonini, M. & Brunori, E. *Hemoglobin and Myoglobin and their Reactions with Ligands*. (North Holland Publishers, 1971).

10 Sasakura, Y. *et al.* Characterization of a direct oxygen sensor heme protein from Escherichia coli. Effects of the heme redox states and mutations at the heme-binding site on catalysis and structure. *J Biol Chem* **277**, 23821-23827 (2002).

11 Hirata, S. *et al.* Characterization of Met95 mutants of a heme-regulated phosphodiesterase from Escherichia coli. *European Journal of Biochemistry* **270**, 4771-4779 (2003).

12 Monson, E. K., Weinstein, M., Ditta, G.S., Helinski, D.R. The FixL protein of *Rhizobium meliloti* can be separated into a heme-binding oxygen sensing domain and a functional C-terminal kinase domain. *Proceedings of the National Academy of Sciences of the United States of America* **89**, 4280-4284 (1992).

13 Rodgers, K. R., Tang, L., Lukat-Rodgers, G. S. & Wengenack, N. L. Insights into the Signal Transduction Mechanism of RmFixL Provided by Carbon Monoxide Recombination Kinetics. *Biochemistry* **40**, 12932-12942 (2001).

14 Wang, J., Stuehr, D. J., Ikeda-Saito, M., Rousseau, D. L. Heme Coordination and Structure of the Catalytic Site in Nitric Oxide Synthase. *Journal of Biological Chemistry* **268**, 22255-22258 (1993).

15 Sono, M., Stuehr, D.J., Ikeda-Saito, M., Dawson, J.H. Identification of Nitric Oxide Synthase as a Thiolate-ligated Heme protein Using Magnetic Circular Dichrosim Spectroscopy. *The Journal of biological chemistry* **270**, 19943-19948 (1995).

16 Marvin, K. A., Reinking, J.L., Lee, A. J., Pardee, K., Krause, H.M., Burstyn, J. N. Nuclear Receptors Homo sapiens Rev-erbbeta and Drsophila melanogaster E75 Are Thiolate-Ligated Heme Proteins Which Undergo Redox-Mediated Ligand Switching and Bind CO and NO. *Biochemistry* **48**, 7056-7071 (2009).

17 Marvin, K. A., Kerby, R.L., Youn, H., Roberts, G.P., Burstyn, J.N. The Transcription Regulator RcoM-2 from Burkholderia xenovorans Is a Cysteine-Ligated Hemoprotein That undergoes a Redox-Mediated Ligand Switch. *Biochemistry* **47**, 9016-9028 (2008).
18 Aono, S. Biochemical and Biophysical Properties of the CO-Sensing Transcriptional Activator CooA. *Accounts of Chemical Research* **36**, 825-831 (2003).
19 Youn, H., Kerby, R. L. & Roberts, G. P. The role of the hydrophobic distal heme pocket of CooA in ligand sensing and response. *The Journal of biological chemistry* **278**, 2333-2340 (2003).
20 Green, E. L., Taoka, S., Banerjee, R., Loehr, T. M. Resonance Raman Characterization of the Hme Cofactor in Cystathione beta-Synthase. Identification of the Fe-S(Cys) Vibration in the Six-Coordinate Low-Spin Heme. *Biochemistry* **40**, 459-463 (2001).
21 Igarashi, J. *et al.* Activation of heme-regulated eukaryotic initiation factor 2alpha kinase by nitric oxide is induced by the formation of a five-coordinate NO-heme complex: optical absorption, electron spin resonance, and resonance raman spectral studies. *The Journal of biological chemistry* **279**, 15752-15762 (2004).
22 Lukat-Rodgers, G. S., Correia, C., Botuyan, M. V., Mer, G. & Rodgers, K. R. Heme-based sensing by the mammalian circadian protein CLOCK. *Inorg Chem* **49**, 6349-6365 (2010).
23 Koudo, R. *et al.* Spectroscopic characterization of the isolated heme-bound PAS-B domain of neuronal PAS domain protein 2 associated with circadian rhythms. *FEBS J* **272**, 4153-4162 (2005).
24 Dioum, E. M. *et al.* NPAS2: a gas-responsive transcription factor. *Science* **298**, 2385-2387 (2002).
25 Smith, A. T. *et al.* Functional divergence of heme-thiolate proteins: a classification based on spectroscopic attributes. *Chemical reviews* **115**, 2532-2558 (2015).
26 Bruno Stefano., F. S., Spyrakis Francesca, Mozzarelli Andrea, Abbruzzetti Stefania, Grandi Elena, Viappiani Cristiano, Feis Alessandro, Mackowiak Stephan, Smulevich Giulietta, Cacciatori Elena, Dominici Paola. The Reactivity with CO of AHb1 and AHb2 from Arabidopsis thaliana is Controlled by the Distal HisE7 and Internal Hydrophobic Cavities. *Journal of the American Chemical Society* **129**, 2880-2889 (2007).
27 Watts, R. A. *et al.* A hemoglobin from plants homologous to truncated hemoglobins of microorganisms. *Proceedings of the National Academy of Sciences of the United States of America* **98**, 10119-10124 (2001).
28 Uzan, J. *et al.* Neuroglobin and other hexacoordinated hemoglobins show a weak temperature dependence of oxygen binding. *Biophysical journal* **87**, 1196-1204 (2004).
29 Ioanitescu, A. I. *et al.* Characterization of nonsymbiotic tomato hemoglobin. *Biophysical journal* **89**, 2628-2639 (2005).
30 Hargrove, M. S. A Flash Photolysis Method to Characterize Hexacoordinate Hemoglobin Kinetics. *Biophysical journal* **79**, 2733-2738 (2000).
31 Smagghe, B. J., Sarath Gautam, Ross Emily, Hilbert Jean-Louis, Hargrove Mark S. Slow Ligand Binding Kinetics Dominate Ferrous Hexacoordiante Hemoglobin Reactivities and Reveal Differences between plants and Other Species. *Biochemistry* **45**, 561-570 (2006).
32 Arredondo-Peter R., H., M.S., Sarath, G., Moran, J.F., Lohrman, J., Olson, J.S., Klucas, R.V. Rice Hemoglobins (Gene Cloning, Analysis, and O2-Binding Kinetics of a Recombinant Protein Synthesized in Escherichia coli). *Plant Physiol.* **1997**, 1259-1266 (1997).
33 Smagghe BJ, S. G., Ross E., Hilbert J-L., Hargrove M. Slow Ligand Binding Kinetics Domainate Ferrous Hexacoordiante Hemoglobin Reactivities and Reveal Differences Between Plants and Other Species. *Biochemistry* **45**, 561-570 (2006).
34 Dewilde, S. *et al.* Biochemical characterization and ligand binding properties of neuroglobin, a novel member of the globin family. *The Journal of biological chemistry* **276**, 38949-38955 (2001).
35 Fago, A., Mathews, A. J., Dewilde, S., Moens, L. & Brittain, T. The reactions of neuroglobin with CO: evidence for two forms of the ferrous protein. *Journal of inorganic biochemistry* **100**, 1339-1343 (2006).

36 Kriegl, J. M. *et al.* Ligand binding and protein dynamics in neuroglobin. *Proceedings of the National Academy of Sciences of the United States of America* **99**, 7992-7997 (2002).
37 Trent III., J. T., Hargrove, M. S. A Ubiquitously Expressed Human Hexacoordinate Hemoglobin. *Journal of Biological Chemistry* **277**, 19538-19545 (2002).
38 Duff, S. M. G., Wittenberg, J.B., Hill, R.D. Expression, Purification, and Properties of Recombinant Barley (*Hordeum sp.*) Hemoglobin. *Journal of Biological Chemistry*, 16746-16752 (1997).
39 Hankeln, T. *et al.* Characterization of Drosophila hemoglobin. Evidence for hemoglobin-mediated respiration in insects. *J Biol Chem* **277**, 29012-29017 (2002).
40 Springer, B. A., Sligar, S. G., Olson, J. S. & Phillips, G. N., Jr. Mechanisms of Ligand Recognition in Myoglobin. *Chemical reviews* **94**, 699-714 (1994).
41 Antonyuk, S. V. *et al.* Carbon monoxide poisoning is prevented by the energy costs of conformational changes in gas-binding haemproteins. *Proceedings of the National Academy of Sciences of the United States of America* **108**, 15780-15785 (2011).
42 Silkstone, G., Jasaitis, A., Wilson, M. T. & Vos, M. H. Ligand dynamics in an electron transfer protein. Picosecond geminate recombination of carbon monoxide to heme in mutant forms of cytochrome c. *J Biol Chem* **282**, 1638-1649 (2007).
43 Lechauve, C. *et al.* Heme Ligand Binding Properties and Intradimer Interactions in the Full-length Sensor Protein Dos from Escherichia coli and Its Isolated Heme Domain. *Journal of Biological Chemistry* **284**, 36146-36159 (2009).
44 Taguchi, S. *et al.* Binding of oxygen and carbon monoxide to a heme-regulated phosphodiesterase from Escherichia coli. Kinetics and infrared spectra of the full-length wild-type enzyme, isolated PAS domain, and Met-95 mutants. *The Journal of biological chemistry* **279**, 3340-3347 (2004).
45 Delgado-Nixon, V. M., Gonzalez, G. & Gilles-Gonzalez, M. A. Dos, a heme-binding PAS protein from Escherichia coli, is a direct oxygen sensor. *Biochemistry* **39**, 2685-2691 (2000).
46 Lechauve, C. *et al.* Heme ligand binding properties and intradimer interactions in the full-length sensor protein dos from Escherichia coli and its isolated heme domain. *J Biol Chem* **284**, 36146-36159 (2009).
47 Gilles-Gonzalez, M. A. *et al.* Heme-Based Sensors, Exemplified by the Kinase FixL, Are a New Class of Heme Protein with Distinctive Ligand Binding and Autoxidation. *Biochemistry* **33**, 8067-8073 (1994).
48 Stone, J. R. & Marletta, M. A. The Ferrous Heme of Soluble Guanylate Cyclase: Formation of Hexacoordinate Complexes with Carbon Monoxide and Nitrosomethane. *Biochemistry* **34**, 16397-16403 (1995).
49 Chang, A. L. *et al.* Phosphodiesterase A1, a Regulator of Cellulose Synthesis in Acetobacter xylinum, Is a Heme-Based Sensor. *Biochemistry* **40**, 3420-3426 (2001).
50 Takahashi, S. *et al.* Heme-heme oxygenase complex: structure and properties of the catalytic site from resonance Raman scattering. *Biochemistry* **33**, 5531-5538 (1994).
51 Sato, A. *et al.* Stationary and time-resolved resonance Raman spectra of His77 and Met95 mutants of the isolated heme domain of a direct oxygen sensor from Escherichia coli. *J Biol Chem* **277**, 32650-32658 (2002).
52 Vogel K. M., S. T. G., Shelver D., Thorsteinsson M. V., Roberts G. P. Resonance Raman Evidence for a Novel Charge Relay Activation Mechanism of the CO-Dependent Heme Protein Transcription Factor CooA. *Biochemistry* **38**, 2679-2687 (1999).
53 Uchida, T., Ishikawa, H., Takahashi, S., Ishimori, K., Morishima, I., Ohkubo, K., Nakajima, H., Aono, S. Heme Enviromental Structure of CooA Is Modulated by the Target DNA Binding. *J. Biol. Chem.* **273**, 19988-19992 (1998).
54 Aono, S. *et al.* Resonance Raman and ligand binding studies of the oxygen-sensing signal transducer protein HemAT from Bacillus subtilis. *The Journal of biological chemistry* **277**, 13528-13538 (2002).
55 Yu, A. E., Hu, S., Spiro, T.G., Burstyn, J.N. Resonance Raman Spectroscopy of Soluble Guanylyl Cyclase Reveals Displacement of Distal and Proximal Heme Ligands by NO. *J. Am. Chem. Soc.* **116**, 4117-4118 (1994).

56 Uchida, T. *et al.* CO-dependent activity-controlling mechanism of heme-containing CO-sensor protein, neuronal PAS domain protein 2. *J Biol Chem* **280**, 21358-21368 (2005).
57 Tsubaki, M., Srivastava, R.B., Yu, N.-T. Resonance Raman Investigation of Carbon Monoxide bonding in (Carbon monoxy) hemoglobin and -myoglobin: Detection of Fe-CO stretching and Fe-C-O Bending Vibrations and Influence of teh Quaternary Structure Change. *Biochemistry* **21**, 1132-1140 (1982).
58 R. Evangelista-Kirkup, G. S., T. G. Spiro. Alternative Carbon Monoxide Binding Modes for Horseradish Peroxidase Studied by Resonance Raman Spectroscopy *Biochemistry* **25**, 4420-4425 (1986).
59 Uno, T., Nishimura, Y., Tsuboi, M. Makino, R., Iizuka, T., Ishimura, Y. . Two types of Conformers with Distinct Fe-C-O Configuration in the Ferrous-CO complex of Horseradish Peroxidase. *The Journal of biological chemistry* **262**, 4549-4556 (1987).
60 A. Feis, J. N. R.-L., R.N.F. Thorneley, G. Smulevich. The Distal Cavity Structure of Carbonyl Horseradish Peroxidase As Probed by the Resonance Raman Spectra of His 42 Leu and Arg 38 Mutants *Biochemistry* **37**, 13575-13581 (1998).
61 Smulevich, G., Evangelista-Kirkup, R., English, A., Spiro, T.G. Raman and Infrared Spectra of Cytochrome *c* Peroxidase-Carbon Monoxide Adducts in Alternative Conformational States. *Biochemistry* **25**, 4426-4430 (1986).
62 Smulevich, G., Mauro, M., Fishel, L. A., English, A.,M., Kraut, J., Spiro, T.G. Cytochrome c Peroxidase Mutant Active Site Structures probed by Resoanance Raman and Infrared Signatures of the CO Adducts. *Biochemistry* **27**, 5486-5492 (1988).
63 Lukat-Rodgers, G. S., Wengenack, N. L., Rusnak, F., Rodgers, K. R. Carbon Monoxide Adducts of KatG and KatG(S315T) as probes of the Heme Site and Isoniazid Binding. *Biochemistry* **40**, 7149-7157 (2001).
64 Kapetanaki, S. M., Chouchane S., Yu, S., Zhao, X., Magliozzo, R. S., Schelvis, J. P. M. . Mycobacterium tuberculosis KatG(S315T) catalase-peroxidase retains all active site properties for proper catalytic function. *Biochemistry* **44**, 243-252 (2005).
65 Tomita, T., Gonzalez, G., Chang, A. L., Ikeda-Saito, M., Gilles-Gonzalez, M-A. A Comparative Resonance Raman Analysis of Heme-Binding PAS Domains: Heme Iron Coordination Structures of the *Bj*FixL, *Ax*PDEA1, *Ec*DOS, and *Mt*Dos Proteins. *Biochemistry* **41**, 4819-4826 (2002).
66 M. Mukai, K. N., H. Nakamura, T. Iizuka, Y. Shiro. Roles of Ile209 and Ile210 on the Heme Pocket Structure and Regulation of Histidine Kinase Activity of Oxygen Sensor FixL from Rhizobium meliloti. *Biochemistry* **39**, 13810-13816 (2000).
67 E. T. Yukl, A., Ioanoviciu, M.M. Nakano, P.R.Ortiz de Montellano, P. Moenne-Loccoz. A Distal Tyrosine Residue Is Required for Ligand Discrimination in DevS from *Mycobacterium Tuberculosis*. *Biochemistry* **47**, 12532-12539 (2008).
68 Tang, K. *et al.* Redox-dependent Ligand Switching in a Sensory Heme-binding GAF Domain of the Cyanobacterium Nostoc sp. PCC7120. *The Journal of biological chemistry* **290**, 19067-19080 (2015).
69 Vogel, K. M., Spiro, T.G. Resonance Raman Evidence for a Novel Charge Relay Activation Mechanism of the CO-Dependent Heme Protein Transcription Factor CooA. *Biochemistry* **38**, 2679-2687 (1999).
70 Coyle, C. M. *et al.* Activation mechanism of the CO sensor CooA. Mutational and resonance Raman spectroscopic studies. *The Journal of biological chemistry* **278**, 35384-35393 (2003).
71 Uchida, T., Ishikawa, H., Takahashi, S., Ishimori, K., Morishima, I., Ohkubo, K., Nakajima, H., Aono, S. Heme Environmental Structure of CooA Is Modulated by the Target DNA Binding. *J. Biol. Chem.* **273**, 19988-19992 (1998).
72 Fan, B., Gupta, G., Danziger, R. S., Friedman, J. M., Rousseau, D.L. Resonance Raman Characterization of Soluble Guanylate Cyclase Expressed from Baculovirus. *Biochemistry* **37**, 1178-1184 (1998).
73 Vogel K. M., H., S., Spiro, T. G., Dierks, E. A., Yu, A. E., Burstyn, J. N. Variable forms of soluble guanylyl cyclase: protein-ligand interactions and the issue of activation by carbon monoxide. *J. Biol. Inorg. Chem.* **4**, 804-813 (1999).

74 Tomita, T., Ogura, T., Tsuyama, S., Imai, Y., Kitagawa, T. Effects of GTP on Bound Nitric oxide of Soluble Guanylate Cyclase Probed by Resonance Raman Spectroscopy. *Biochemistry* **36**, 10155-10160 (1997).
75 Deinum, G., Stone, J. R., Babcock, G. T., Marletta, M. A. . Binding of Nitric oxide and Carbon Monoxide to Soluble Guanylate Cyclase As observed with Resonance Raman Spectroscopy *Biochemistry* **35**, 1540-1547 (1996).
76 Igarashi, J., Sato, A., Kitagawa, T., Sagami, I. & Shimizu, T. CO binding study of mouse heme-regulated eIF-2α kinase: kinetics and resonance Raman spectra. *Biochimica et Biophysica Acta (BBA) - Proteins and Proteomics* **1650**, 99-104 (2003).
77 Ishikawa, H., Yun, B-G., Takahashi, S., Hori, H., Matts, R. L., Ishimori, K., Morishima, I. NO-Induced Activation Mechanism of the Heme-Regulated eIF2a Kinase. *J. Amer. Chem. Soc.* **124**, 13696-13697 (2002).
78 Abbruzzetti, S. *et al.* Heterogeneous kinetics of the carbon monoxide association and dissociation reaction to nitrophorin 4 and 7 coincide with structural heterogeneity of the gate-loop. *Journal of the American Chemical Society* **134**, 9986-9998 (2012).
79 K.A. Marvin, J. L. R., A. J. Lee, K. Pardee, H.M. Krause, J.N. Burstyn. Nuclear Receptors Homo Sapiens Rev-erbbeta and Drosophila melanogaster E75 Are Thiolate-Ligated Heme Proteins Which Undergo Redox-Mediated Ligand Switching and Bind CO and NO *Biochemistry* **48**, 7056-7071 (2009).
80 Andrew, C. R., Green, E. L., Lawson, D. M., Eady, R. R. Resonance Raman Studies of Cytochrome c' Support the Binding of NO and CO to Opposite Sides of the Heme: Implications for Ligand Discrimination in Heme-Based Sensors. *Biochemistry* **40**, 4115-4122 (2001).
81 Wang, J., Stuehr, D. J., Rousseau, D. L. Interactions between Substrate Analogues and Heme Ligands in Nitric oxide Synthase. *Biochemistry* **36**, 4595-4606 (1997).
82 Fan, B., Wang, J., Stuehr, D.J., Rousseau, D.L. NO Synthase Isozymes Have Distinct Substrate Binding Sites. *Biochemistry* **36**, 12660-12665 (1997).
83 Li, D., Stuehr, D. J., Yeh, S. R. & Rousseau, D. L. Heme distortion modulated by ligand-protein interactions in inducible nitric-oxide synthase. *The Journal of biological chemistry* **279**, 26489-26499 (2004).
84 Wells, A., Li, P., Champion, P. M. Resonance Raman Investigations of *Escherichia coli*-Expressed *Pseudomonas putida* Cytochrome P450 and P420. *Biochemistry* **31**, 4384-4393 (1992).
85 Uno, T., Nishimura, Y., Makino, R., Iizuka, T., Ishimura, Y., Tsuboi, M. The Resonance Raman Frequencies of the Fe-CO Stretching and Bending Modes in the CO Complex of Cytochrome P-450cam. *J. Biol. Chem.* **260**, 2023-2026 (1985).
86 O'Keefe, D. H., Ebel, R. E., Peterson, J. A. Maxwell, J. C., Caughey, W. S. . An Infrared Spectroscopic Study of Carbon Monoxide Bonding to Ferrous Cytochrome P-450. *Biochemistry* **17**, 5845-5852 (1978).
87 Jung, C., Hui Bon Hoa, G., Schroeder, K-L., Simon, M., Doucet, J. P. Substrate Analogue Induced Changes of the CO-Stretching Mode in the Cytochrome P450cam-Carbon Monoxide Complex. *Biochemistry* **31**, 12855-12862 (1992).
88 Hu, S., Kincaid, J. R. Resonance Raman Characterization of Nitric Oxide Adducts of Cytochrome P450cam: The Effect of Substrate Structure on the Iron-Ligand Vibrations. *J. Amer. Chem. Soc.* **113**, 2843-2850 (1991).
89 Nagano, S., Shimada, H., Tarumi, A. Hishiki, T., Kimata-Ariga, Y., Egawa, T., Suematsu, M., Park, S-Y., Adachi, S-i., Shiro, Y., Ishimura, Y. Infrared Spectroscopic and Mutational Studies on Putidaredoxin-Induced Conformational Changes in Ferrous CO-P450cam. *Biochemistry* **42**, 14507-14514 (2003).
90 Unno, M. *et al.* Complex formation of cytochrome P450cam with Putidaredoxin. Evidence for protein-specific interactions involving the proximal thiolate ligand. *The Journal of biological chemistry* **277**, 2547-2553 (2002).

91 Mak, P. J., Gregory, M. C., Sligar, S. G. & Kincaid, J. R. Resonance Raman spectroscopy reveals that substrate structure selectively impacts the heme-bound diatomic ligands of CYP17. *Biochemistry* **53**, 90-100 (2014).
92 Vogel, K. M., Kozlowski, P.M., Zgierski, M. Z., Spiro, T.G. Role of the axial ligand in heme-CO backbonding;DFT analysis of vibrational data. *Inorganic Chimica Acta* **297**, 11-17 (2000).
93 Ray, G. B., Li, X-Y., Ibers, J. A., Sessler, J. L., Spiro, T.G. How Far Can Proteins Bend the FeCO Unit? Distal Polar and Steric Effects in Heme Proteins and Models. *J. Am. Chem. Soc.* **116**, 162-176 (1994).
94 Kerr, E. A., Mackin, H. C., Yu, N-T. Resonance Raman Studies of Carbon Monoxide Binding to Iron 'Picket Fence' Porphyrin with Unhindered and Hindered Axial Bases. An Inverse Relationship between Binding Affinity and the Strength of Iron-Carbon Bond. *Biochemistry* **22**, 4373-4379 (1983).
95 Wayland, B. B., Mehne, L.F., Swartz, J. Mono- and Biscarbonyl Complexes of iron(II) Tetraphenylporphyrin. *J. Am. Chem. Soc.* **100**, 2379-2383 (1978).
96 Nicoletti, F. P., Howes, B.D., Fittipaldi, M., Fanali, G., Fasano, M., Ascenzi, P., Smulevich, G. Ibuprofen Induces an Allstoric Conformational Transition in the Heme Complex of Juman Serum Albumin with Sigmificant Effects on Heme Ligation. *J. Am. Chem. Soc.* **130**, 11677-11688 (2008).
97 Xe, X., Yu, A., Georgiev, G.Y., Gruia, F., Ionascu, D., Cao, W., Sage, J.T., Champion, P. M. CO Rebinding to Protoheme:Investigations of the Proximal and Distal Contributions to the Geminate Rebinding Barrier. *J. Am. Chem. Soc.* **127**, 5854-5861 (2005).
98 Lobato, L. *et al.* Dynamics of the heme-binding bacterial gas-sensing dissimilative nitrate respiration regulator (DNR) and activation barriers for ligand binding and escape. *The Journal of biological chemistry* **289**, 26514-26524 (2014).
99 Benabbas, A., Karunakaran, V., Youn, H., Poulos, T. L. & Champion, P. M. Effect of DNA binding on geminate CO recombination kinetics in CO-sensing transcription factor CooA. *The Journal of biological chemistry* **287**, 21729-21740 (2012).
100 Kumazaki, S. *et al.* Dissociation and recombination between ligands and heme in a CO-sensing transcriptional activator CooA. A flash photolysis study. *The Journal of biological chemistry* **275**, 38378-38383 (2000).
101 Liebl, U., Bouzhir-Sima, L., Kiger L., Marden M. C., Lambry J-C., Negrerie M., Vos M. H. Ligand Binding Dynamics to the Heme Domain of the Oxygen Sensor Dos from *Escherichia coli*. *Biochemistry* **42**, 6527-6535 (2003).
102 Jasaitis, A., Hola, K., Bouzhir-Sima, L., Lambry, J-C., Balland, V., Vos, M.H., Liebl, U. Role of Distal Arginine in Early Sensing Intermediates in the Heme Domain of the Oxygen Sensor FixL. *Biochemistry* **45**, 6018-6026 (2006).
103 Bouzhir-Sima, L., Motterlini, R., Gross, J., Vos, M. H. & Liebl, U. Unusual Dynamics of Ligand Binding to the Heme Domain of the Bacterial CO Sens or Protein RcoM-2. *The journal of physical chemistry. B* (2016).
104 Negrerie, M., Bouzhir, L., Martin, J. L. & Liebl, U. Control of nitric oxide dynamics by guanylate cyclase in its activated state. *The Journal of biological chemistry* **276**, 46815-46821 (2001).
105 Kruglik, S. G. *et al.* Molecular basis for nitric oxide dynamics and affinity with Alcaligenes xylosoxidans cytochrome c. *The Journal of biological chemistry* **282**, 5053-5062 (2007).
106 Silkstone, G., Kapetanaki, S. M., Husu, I., Vos, M. H. & Wilson, M. T. Nitric oxide binds to the proximal heme coordination site of the ferrocytochrome c/cardiolipin complex: formation mechanism and dynamics. *The Journal of biological chemistry* **285**, 19785-19792 (2010).
107 S. Taoka, R. B. Characterization of NO binding to human cystathione beta-synthase: possible implications of the effects of CO and NO binding to the human enzyme. *Journal of inorganic biochemistry* **87**, 245-251 (2001).
108 Reynolds, M. F. *et al.* Electronic Absorption, EPR, and Resonance Raman Spectroscopy of CooA, a CO-Sensing Transcription Activator from R. rubrum, Reveals a Five-Coordinate NO-Heme. *Biochemistry* **39**, 388-396 (2000).

109 Yoshimura, T. *et al.* Spectral properties of nitric oxide complexes of cytochrome c' from Alcaligenes sp. NCIB 11015. *Biochemistry* **25**, 2436-2442 (1986).
110 Gunn, A., Derbyshire, E. R., Marletta, M. A. & Britt, R. D. Conformationally distinct five-coordinate heme-NO complexes of soluble guanylate cyclase elucidated by multifrequency electron paramagnetic resonance (EPR). *Biochemistry* **51**, 8384-8390 (2012).
111 Petrich, J. W., Poyart, C., Martin, J.L. Photophysics and Reactivity of Heme Proteins: A Femtosecond Absorption Study of Hemoglobin, Myoglobin, and Protoheme. *Biochemistry* **27**, 4049-4060 (1988).
112 Franzen, S., Kiger, L., Poyart, C., Martin, J-L. Heme Photolysis Occurs by Ultrafast Excited State Metal-to-Ring Charge Transfer. *Biophysical journal* **80**, 2372-2385 (2001).
